# Supplementary material for: Kcnj16 (Kir5.1) Gene Ablation Causes Subfertility and Increases the Prevalence of Morphologically Abnormal Spermatozoa
Source: Int J Mol Sci. 2021 Jun 1;22(11):5972. doi: 10.3390/ijms22115972 (PMC8199489; doi:10.3390/ijms22115972)
Supplement: Supplementary file 1 [file ijms-22-05972-s001.zip › ijms-1218231-supplementary.pdf]

Supplementary Material

Kcnj16 (*Kir5.1*) Gene Ablation Causes Subfertility and Increases the Prevalence of Morphologically Abnormal Spermatozoa

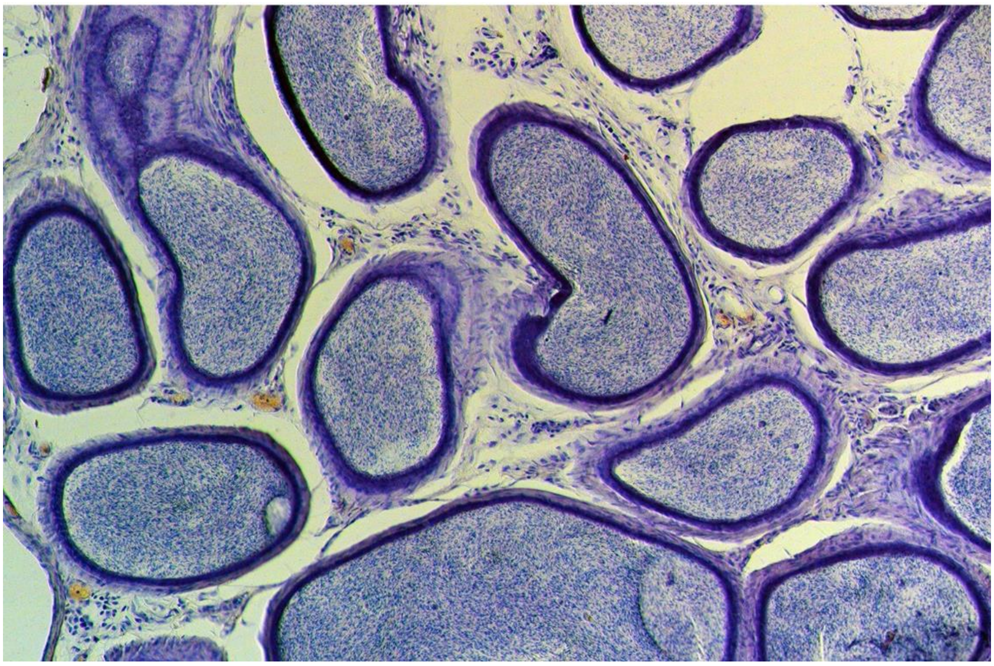

Figure S1. Hematoxylin and eosin staining of cauda epididymis. Sample image showing the typical histological structures of epididymis collected from a WT mouse, as well as the numerous spermatozoa located in the lumen.
